# Subdimensions of social‐communication behavior in autism—A replication study

**DOI:** 10.1002/jcv2.12077

**Published:** 2022-05-04

**Authors:** Sanna Stroth, Hauke Niehaus, Nicole Wolff, Luise Poustka, Veit Roessner, Inge Kamp‐Becker, Dominik Endres

**Affiliations:** ^1^ Department of Child and Adolescent Psychiatry, Psychosomatics and Psychotherapy Philipps University Marburg Germany; ^2^ Department of Psychology Philipps University Marburg Marburg Germany; ^3^ Department of Child and Adolescent Psychiatry Medical Faculty of the TU Dresden Dresden Germany; ^4^ Department of Child and Adolescent Psychiatry and Psychotherapy University Medical Center Göttingen Göttingen Germany

**Keywords:** autism, Bayesian exploratory factor analysis (BEFA), interaction quality, social communication impairment, subdimensions

## Abstract

**Introduction:**

In order to identify more refined dimensions of social‐communication impairments in autism spectrum disorder (ASD) a previous study applied exploratory and confirmatory factor analyses to diagnostic algorithm scores of the autism diagnostic observation schedule (ADOS), Module 3. A three‐factor model consisting of repetitive behaviors, impairments in ‘Basic Social‐Communication’ and in ‘Interaction quality’ (IQ) was established and confirmed. The current study aimed to replicate this model in an independent sample. To advance our understanding of the latent structure of social communication deficits, previous work was complemented by a probabilistic approach.

**Methods:**

Participants (*N* = 1363) included verbally fluent children and young adults, diagnosed as ASD or non‐ASD based on “gold standard” best‐estimate clinical diagnosis. Confirmatory factor analysis examined the factor structure of algorithm items from the ADOS Module 3 and correlations with individual characteristics (cognitive abilities, age) were analyzed. Linear Regressions were used to test the contribution of each latent factor to the prediction of an ASD diagnosis. To tackle large inter‐correlations of the latent factors, a Bayesian exploratory factor analysis (BEFA) was applied.

**Results:**

Results confirmed the previously reported observation of three latent dimensions in the ADOS algorithm reflecting ‘Restricted, Repetitive Behaviors’, ‘Basic Social‐Communication’ behaviors and ‘Interaction Quality’. All three dimensions contributed independently and additively to the prediction of an ASD diagnosis.

**Conclusion:**

By replicating previous findings in a large clinical sample our results contribute to further conceptualize the social‐communication impairments in ASD as two dimensional.

1

**Key points**

1. In a large clinical sample of verbally fluent children and adolescents with and without ASD, we could replicate the finding of two separable subdimensions of social‐communication impairment
2. A Bayesian exploratory factor analysis supports the conceptualization of social‐communication impairments in ASD as two‐ or even multi‐dimensional
3. The “Basic SOC” subdomain represents a replicable and stable factor that seems appropriate as an index of ASD symptom severity
4. Identifying subdimensions of social‐communication impairment and subtypes along the autism spectrum may support future research efforts trying to link neurobiological mechanisms to specific types of behaviors

## 2 INTRODUCTION

The concept of autism spectrum disorder (ASD) has shifted from a “childhood condition” with associated challenges in language and intellectual functioning, to a wider concept of ASD including individuals with only mild symptoms/autistic traits or those who do not show symptoms until later in life (Vivanti & Messinger, 2021). According to the diagnostic conceptualizations in the diagnostic and statistical manual of mental disorders fourth ed (American Psychiatric Association, 1994) as well as in the international statistical classification of diseases, 10th revision (World Health Organization [WHO], 1993), ASD used to be conceptualized as a multi‐categorical disorder with different subtypes (childhood autism, asperger syndrome, pervasive developmental disorder not otherwise specified or atypical autism and others). However, these ASD subtypes could not be differentiated as distinct, empirically defined subgroups (Lord et al., 2012a; Walker et al., 2004) but rather overlapping conditions (Macintosh & Dissanayake, 2004; Snow & Lecavalier, 2011). Consequently, ASD is now conceptualized as a hybrid model in the diagnostic and statistical manual of mental disorders, fifth ed. (DSM‐5) (American Psychiatric Association, 2013): Dimensional individual differences in symptom severity and general impairment are considered within a categorical umbrella term of ASD (Frazier et al., 2012; Grzadzinski, Huerta, & Lord, 2013). The development towards a dimensional understanding and the broadening of the diagnostic criteria has led to substantial and increasing heterogeneity in the clinical phenotype of ASD (Fombonne, 2020; Hansen, Schendel, & Parner, 2015). This heterogeneity is a challenging problem in research as well as in clinical practice (Lord et al., 2020; Mottron & Bzdok, 2020). The discovery of valid biomarkers is hampered by phenotypic heterogeneity, symptom overlap with other conditions as well as co‐occurring conditions in ASD. This is visible in a drop of effect sizes by up to 80% from cognitive, electroencephalogram and neuroanatomical group comparison studies in the past 2 decades (Rødgaard, Jensen, Vergnes, Soulières, & Mottron, 2019).

In order to define the boundaries of ASD many efforts have been made to identify symptom dimensions which has led to the postulation of two symptom domains (social communication and restricted interests and/or repetitive behaviors) in the DSM‐5 criteria for ASD. However, research could not yet identify replicable subdomains within the social‐communication domain that may define subgroups of individuals with ASD. Bishop and colleagues argue that more refined dimensions of the social‐communication domain of ASD are needed “to elucidate the clinical, nosological and biological boundaries of the multiple disorders associated with social‐communication impairment” (Bishop et al. 2016; p. 909). They examined the organizational structure of clinician‐observed social‐communication deficits with exploratory factor analysis in a sample of 238 children with and without ASD between the ages of 2 and 12 years and found a three‐factor model consisting of restricted, repetitive behaviors (RRB) and two separate social‐communication behavior dimensions, “Basic Social‐Communication” (Basic SOC) and “Interaction Quality”(IQ). This factor structure could be replicated by confirmatory factor analysis (CFA) in an independent sample. The impairments in “Basic SOC” behaviors included items measuring eye contact, facial expressions, gestures and shared enjoyment and were separated from impairments in “Interaction Quality”, including items measuring conversation, amount of reciprocal social communication, overall quality of rapport, and quality of social response. While scores in “Interaction Quality” were significantly associated with nonverbal Intelligence quotient (IQ) and male sex in the ASD group and with age in the non‐ASD group, scores in “Basic SOC” were not significantly associated with these phenotypic variables but “remarkably intact in children who do not have ASD, even in the presence of significant other impairments or risk factors” (Bishop et al., 2016, p. 913). The authors conclude that basic impairments in nonverbal communication and shared affect seem to be specific to ASD and thus could provide a more specific index of ASD severity, whereas impairments in interaction quality appear to be less specific to ASD and more impacted by other child characteristics and thus may be more relevant for differential diagnoses.

The current study aimed to replicate this suggested two‐fold nature of social‐communication impairments in a large and independent sample of individuals with ASD and a large sample of individuals with relevant differential diagnoses and other developmental issues. We address two hypotheses originating from the results of Bishop et al. (2016): 1) child characteristics should not be associated with the basic dimension and 2) the factor “Basic SOC” would be more predictive of ASD than “Interaction Quality”. We thus tested correlations of age and verbal IQ with the latent subdimensions of social communication in the current sample. Lastly, in order to identify the model that best fit our current data, the methodological approach was complemented by a Bayesian exploratory factor analysis (BEFA).

## 3 METHOD

### 3.1 Participants

The data used for the present study represent a subsample extracted from an existing research database of the ASD‐Net, a state‐funded research network (Kamp‐Becker et al., 2017). Datasets stem from individuals who had been referred to ASD specialized outpatient clinics for a diagnostic assessment due to a suspicion of ASD. To assemble a representative sample of individuals who seek an investigation of ASD, the presence of a clinical suspicion of ASD was the general inclusion criterion. Subjects were either diagnosed as having ASD or the diagnosis was excluded (non‐ASD) based on “gold standard” best‐estimate clinical diagnosis (BEC) by autism diagnostic observation Schedule ADOS‐trained examiners, who attended standard research trainings and maintained research reliability with project consultants through (semi)‐annual workshops and video scoring and research reliable ADOS experts for supervision. BEC diagnoses rely on the evaluation of two clinicians after extensive examination and review of all available information from the participant's record (IQ, neuropsychological testing, reports from other institutions, school reports, home videos, ADOS, ADI‐R, differential diagnostic examination with established structured questionnaires and structural clinical interviews) according the German guidelines for ASD and based on the international statistical classification of diseases and related health problems 10th revision (ICD‐10). Within the ASD group, different ICD‐10 autism spectrum diagnoses (e.g. Autism, Asperger Syndrome) with or without co‐occurring conditions were grouped together. The non‐ASD group consisted of individuals with other mental disorders (e.g. anxiety disorders, attention deficit hyperactivity disorder) and other developmental issues. Clinical characteristics of the non‐ASD sample are presented in Table 1.

**TABLE 1:** Frequencies of ICD‐10 (axis 1 and axis 2) disorders in the non‐ASD group (multiple diagnoses per individual possible)

	Frequency (%)
Disorders axis 1	
Attention‐deficit (Hyperactivity) disorders (F90, F98.8)	289 (35.8)
Conduct disorders (F91, F92)	94 (11.6)
Hyperkinetic conduct disorder (F90.1)	93 (11.5)
Emotional disorders with onset specific to childhood (F93)	95 (11.8)
Disorders of social functioning with onset specific to childhood and adolescence (F94)	75 (9.3)
Other behavioral and emotional disorders with onset usually occurring in childhood and adolescence (F98.0–6)	69 (8.5)
Tic disorder (F95)	30 (3.7)
Obsessive‐compulsive disorder (F42)	7 (0.9)
Phobic and anxiety disorder (F40, 41)	8 (1.0)
Depressive disorder (F32, 34)	3 (0.4)
Others	54 (7.7)
Disorders axis 2 (no axis 1 diagnosis)	
Specific developmental disorders of speech and language (F80)	81 (9.9)
Specific developmental disorders of scholastic skills (F81)	52 (6.3)
Specific developmental disorder of motor function (F82)	73 (9.0)
Mixed specific developmental disorders (F83)	20 (2.5)
Other and unspecified disorders of psychological development (F88, 89)	7 (0.9)
Without any diagnosis of mental disorders	175

Frequencies refer to *N* = 806 non‐ASD individuals.

Participants' data were collected retrospectively from the respective medical record and analyzed anonymously. The procedure was approved by the local ethics committee (Az. 92/20) and due to the retrospective nature of data collection and analysis of anonymized data, the need for informed consent was waived by the ethics committee. All methods were performed in accordance with the relevant institutional and international research guidelines and regulations.

The study included 1363 datasets (4–27 years of age, *n* = 557 with ASD, 9.5% female, *n* = 806 with other mental disorders, 12.8% female), all evaluated with the ADOS Module three during the diagnostic process.

IQ scores, including full scale IQ, verbal IQ and non‐verbal IQ, were available for 902 participants (82.1%). A statement regarding the intellectual level from the clinical record (average, above average, borderline, mildly impaired, etc. following ICD‐10 categories) was available for another 6 cases (6.2%). Preliminary analyses on group differences regarding age and IQ and ASD symptoms are reported in Table 2.

**TABLE 2:** Sample description

	Non‐ASD	ASD	*t*‐Value	*p*	*d*
N	806	557			
Age	9.9 (2.6)	10.4 (2.8)	3.3	0.001	0.17
Full scale IQ	98.5 (18.5)	97.2 (18.3)	1.1	0.26	0.07
Verbal IQ	101.8 (18.8)	100.8 (19.6)	0.7	0.16	0.05
Non‐verbal IQ	99.6 (18.8)	96.5 (18.2)	2.6	0.009	0.16
SA	3.0 (3.4)	9.5 (4.5)	29.9	<0.001	**1.63**
RRB	0.3 (0.6)	1.4 (1.3)	20.1	<0.001	**1.09**
CSS	2.1 (1.8)	5.7 (2.4)	10.7	<0.001	**1.70**

SA = Social Affect Scale, RRB = Restricted, repetitive behaviors scale, CSS = Calibrated Severity Score from the ADOS. Numbers in brackets present Standard Deviations (SD). *d* = Cohen's effect size (0.2–0.5 = small effect, 0.5–0.8 = medium effect, >0.8 = large effect). Bold numbers indicate effects exceeding the threshold of large effects.

Abbreviations: ADOS, autism diagnostic observation schedule; IQ, intelligence quotient.

### 3.2 Measures

The German versions of the autism observation schedule (ADOS and ADOS‐2; Poustka et al., 2015; Rühl, Bölte, Feineis‐Matthews, & Poustka, 2004) were administered. The ADOS is a semi‐structured and standardized observation tool which is part of the established “gold standard” to diagnose ASD (National Collaborating Center for Women's and Children's Health [UK], 2011). The ADOS consists of four modules (plus a toddler module in ADOS‐2), one of which is selected depending the individual's level of expressive language, chronological age and the appropriateness of assessment materials. It comprises a semi‐structured interaction of the participant with a clinically‐trained administrator to capture important social‐communicative behaviors as well as stereotypic and repetitive behavioral features. In Module 3, which is intended for verbally fluent children and adolescents, these aspects are coded via 29 items. Codes fall on an ordinal scale from 0 (“when the behavior shows no evidence of abnormality as specified”), 1 (“when the behavior is mildly abnormal or slightly unusual but not necessarily grossly abnormal or not as clear as the type specified”) and 2 (“when the behavior is definitely abnormal in the way specified”) to 3 (“when the behavior is markedly abnormal in a way that interferes with the assessment or when the behavior is so limited that judgment about quality are impossible”), with additional codes of 7 and 8 for abnormal behavior or behavior not exhibited during the observation, and a code of 9 for missing values (i.e. answers omitted or left blank; Lord et al., 2012b). The behavioral items are grouped into two respective three domains: communication, reciprocal social interaction – which are combined to social affect (SA) in ADOS‐2 – and RRB. The ADOS‐2 diagnostic algorithm in module three comprises 14 items and allows for classifications of autism and autism spectrum versus non‐ASD according to respective cut‐offs.

### 3.3 Statistical analyses

Following ADOS conventions codes of seven and eight were re‐coded to 0. Codes of three were re‐coded to 2. Missing values were excluded listwise (12 cases had missing values in the ADOS data und were thus excluded from CFA and BEFA). Group characteristics were explored via *t*‐Tests. In order to test the latent dimensions suggested by Bishop et al. (2016), a CFA was performed with a robust weighted least square mean and variance adjusted estimator and oblique rotation (goemin) to allow factor correlations. Factor number and factor‐item‐assignment was based on the model of Bishop et al. (2016) including three factors (“Basic SOC”, ‘“Interaction Quality” and ‘“RRB”). The ADOS item “quality of social overtures” (QSOV) was excluded from the CFA following the exclusion by Bishop et al. (2016). Model fit was assessed by a combination of parameters: The goodness‐of‐fit index χ^2^ is sensitive to sample size and was therefore combined with the root‐mean‐square error of approximation (RMSEA), the comparative fit index (CFI) and the Tucker‐Lewis index (TLI). Evaluation of model fit was based on criteria used by Bishop et al. (2016) (fit of RMSEA cuf‐offs of 0.01 (excellent), 0.05 (good), and 0.08 (acceptable; MacCallum, Browne, & Sugawara, 1996) and CFI ≥0.96 and TLI ≥0.95 for good fit (Hu & Bentler, 1999). The model fit of the three factor model was compared to a two‐dimensional model of ASD symptoms consisting of a social‐communication factor and an RRB factor as suggested by the DSM‐5.

Due to the observation that the latent factors were substantially correlated and a CFA model containing only one social‐communication factor showed similar quality indices as the two‐factor model, analyses were complemented by BEFA. Owing to the exploratory nature of this analysis, the ADOS item “Quality of Social Overtures” (QSOV) was re‐entered into the set of items. Through its probabilistic assumptions, BEFA can address factor extraction and parameter estimation in one step rendering the sample split for exploratory and subsequent confirmatory analyses unnecessary (Conti, Frühwirth‐Schnatter, Heckman, & Piatek, 2014). Additionally, BEFA circumvents prominent ad‐hoc conventions for factor extraction by producing intuitive estimates of posterior model probability. BEFA is a dedicated factor‐model allowing manifest variables to load only on a single factor but permits extracted latent factors to be correlated. The BEFA model estimates the posterior probability for the number of latent factors, the idiosyncratic variances, the covariance matrix of the latent factors, the factor‐loading matrix and the indicator matrix. Model parameters are estimated via Markow‐Chain‐Monte‐Carlo method (Metropolis‐Hastings). Bayesian methods address the challenge of reasoning under uncertainty (e.g. finding the optimal model among candidate models) by modeling parameters as probability distributions (Eddy, 2004) and producing consistent and intuitive estimates of posterior probabilities. Data are only discarded from the model if they have an exceedingly small probability (≤0.02) of loading onto any factor (Conti et al., 2014). The current study used the R package BayesFM (Piatek, 2019). A sparse model with uninformative priors (Huang & Wand, 2013) was specified as to estimate the optimal data‐driven factor solution. Following a predictive analyses for the prior specification for the number of latent factors and the covariance matrix of the latent factors, the factor model was estimated using the parameters presented in the supporting information (Table S1). All other parameters were set to default values (see BayesFM documentation for details).

Associations of child characteristics (age, sex, IQ) with the three symptom dimensions were examined via Pearson's correlation in both groups (ASD and non‐ASD).

Via a logistic regression, the contribution of the three dimensions to the prediction of ASD versus non‐ASD diagnoses, replicating the approach used by Bishop et al. (2016) was examined. A second logistic regression was conducted to investigate the contribution of the BEFA factors to the prediction of ASD versus non‐ASD diagnoses. Child characteristics (age, IQ and sex) were controlled for. Items and their abbreviations are listed in Table S2.

## 4 RESULTS

### 4.1 Testing the factor model

Confirmatory factor analyses were used to replicate the factor structure suggested by Bishop et al. (2016). We found a three‐dimensional model with acceptable model fit and a two‐dimensional model with inferior but still acceptable model fit. Figure S1 in the supporting information materials depicts the structure of the models with two und three latent factors.

Model fit indices from the three‐factor model were as follows: χ^2^(51) = 266.2, *p* < 0.001, CFI = 0.983, TLI = 0.978 RMSEA = 0.056. The Basic SOC‐factor correlated high with the Interaction Quality‐factor (*r* = 0.92, *p* < 0.001) and even in a high range with the RRB‐factor (*r* = 0.72, *p* < 0.001). The Interaction Quality‐factor also correlated in a high range with the RRB‐factor (*r* = 0.71, *p* < 0.001).

A two‐factor model also showed acceptable fit indices: χ^2^(53) = 298.4, *p* < 0.001, CFI = 0.981, TLI = 0.976 RMSEA = 0.059. The communication and interaction‐factor correlated high with the RRB‐factor (*r* = 0.73, *p* < 0.001). Factor loadings are reported in Table 3.

**TABLE 3:** Results from the confirmatory factor analysis: factor loadings for the three factor solution and two factor solution

	Three factor solution			Two factor solution	
	Basic SOC	Int. Qual.	RRB	SA	RRB
Social affect					
Descriptive, conventional, instrumental, or informational gestures	0.701			0.581	
Unusual eye contact	0.626			0.455	
Facial expressions directed to others	0.810			0.683	
Shared enjoyment in interaction	0.819			0.694	
Amount of reciprocal social communication		0.908		0.814	
Conversation		0.860		0.772	
Overall quality of rapport		0.787		0.668	
Quality of social response		0.821		0.701	
RRB					
Stereotyped/Idiosyncratic use of words or phrases			0.727		0.601
Excessive interest in or references to unusual or highly specific topics or objects or repetitive behaviors			0.655		0.393
Hand and finger and other complex mannerisms			0.561		0.389
Unusual sensory interest in play material/Person			0.553		0.438

Basic SOC = basic social communication; Int. Qual. = Interaction Quality; RRB = restricted and repetitive behaviors; SA = Social Affect; Parameters of factor loadings are standardized.

Comparison of the models via a Chi squared difference test showed superiority of the three‐factor model over the two‐factor model (χ^2^_diff_ = 32.12; df = 2, *p* < 0.001), although differences in the fit indices seemed negligible.

### 4.2 Bayesian exploratory factor analysis

**FIGURE 1:**
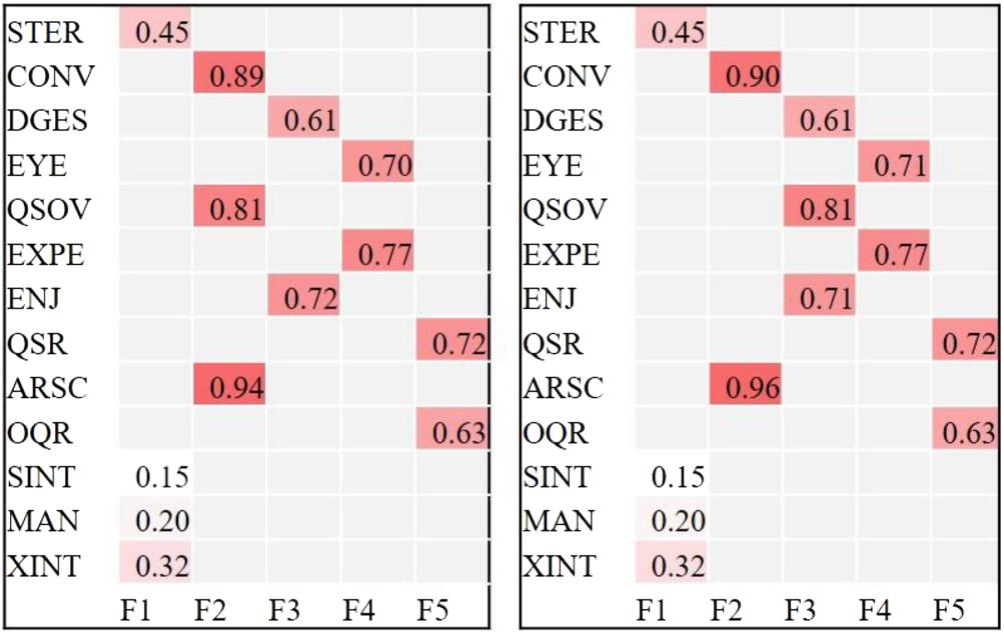
Figure 1 shows the structure and factor loadings of the two 5 factor models including the Autism Diagnostic Observation Schedule (ADOS) item Quality of Social Overtures (QSOV)

The specified BEFA model returned a posterior probability of 85.1% for a 5‐factor model. Two almost identical models with 5 factors showed posterior probabilities of pmp_m1_ = 49.3% and pmp _m2_ = 35.8%. Figure 1 shows the factor structure of both five factor models. Factor solutions with less factors were unlikely given the observed data (*K* = 3, pmp = 9.4%, *K* = 4, pmp = 5.5%).

We had re‐entered the ADOS algorithm item “Quality of Social Overtures” (QSOV) back into the analysis after it had been discarded by Bishop et al. due to unclear factor loadings on the Basic SOC and interaction quality subdimensions. It was argued that this items seems to capture aspects of behaviors that rely on skills in both dimensions (see Bishop et al., 2016, p. 912). In our BEFA analysis this items again switched between both subdimensions loading on a factor related to basic SOC in m1 and on a factor related to interaction quality in m2 indicating that indeed a clear assignment to one factor is inadequate. We thus decided exclude QSOV from the final BEFA model.

**FIGURE 2:**
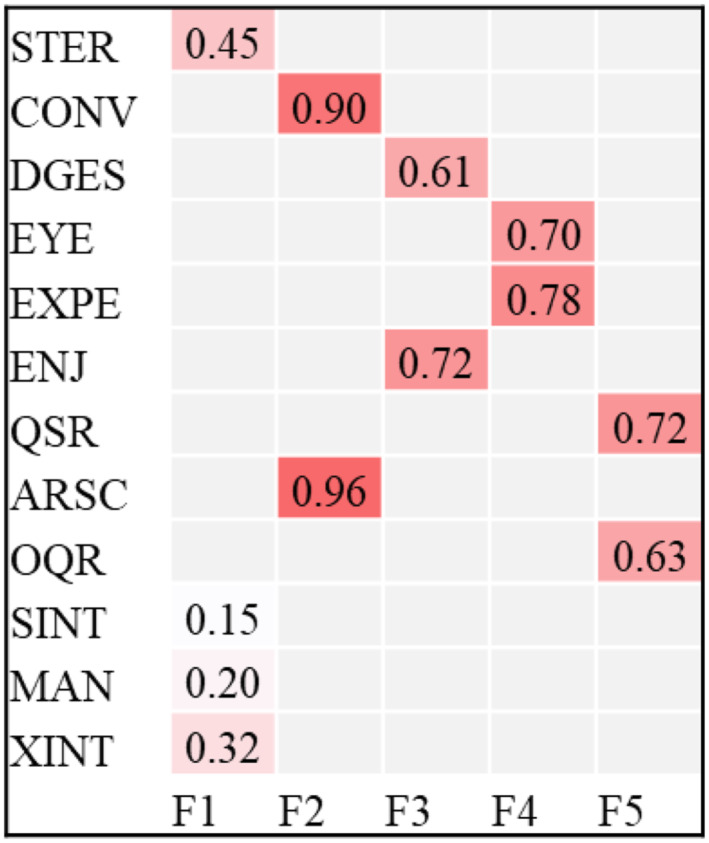
Shows the structure and factor loadings of the final Bayesian exploratory factor analysis (BEFA) five‐factor model

The final BEFA model returned a 5‐factor model with a posterior model probability of pmp = 100% meaning that models with less factors were unlikely in our data. Figure 2 depicts the final BEFA model. The posterior model parameters of the factor loadings and their idiosynchratic variances for each ADOS item are shown in Table 4. None of the manifest variables had a high posterior probability of being discarded so all manifest variables loaded on a factor.

**TABLE 4:** Factor loadings and idiosynchratic variances for each ADOS item in the Bayesian exploratory factor analysis (BEFA) for a four‐factor model and a five factor model

5‐Factor model (pmp = 100%)					
	Dedic	Mean factor loading	sd	Idiosynchratic variances	sd
STER	1	0.454	0.016	0.169	0.010
CONV	2	0.899	0.021	0.188	0.010
DGES	3	0.609	0.019	0.292	0.012
EYE	4	0.699	0.025	0.568	0.024
EXPE	4	0.776	0.019	0.133	0.012
ENJ	3	0.719	0.020	0.230	0.011
QSR	5	0.721	0.018	0.144	0.009
ARSC	2	0.957	0.021	0.129	0.009
OQR	5	0.630	0.017	0.180	0.009
**SINT**	1	0.151	0.010	0.089	0.004
**MAN**	1	0.202	0.013	0.151	0.006
XINT	1	0.318	0.015	0.194	0.009

pmp = posterior model probability; dedic = dedicated factor the manifest variable loads on. mean = mean factor loading across MCMC samples. Bold font indicates a change in dedicated factor.

Abbreviation: ADOS, autism diagnostic observation schedule; ARSC, Amount of Reciprocal Social Communication; CONV, Conversation; DGES, Descriptive, Conventional, Instrumental, or Informational Gestures; ENJ, Shared Enjoyment in Interaction; EXPE, Facial Expressions Directed to Examiner; EYE, Unusual Eye Contact; MAN, hand and finger and other complex mannerisms; MCMC, Markow‐Chain‐Monte‐Carlo method; OQR, Overall Quality of Rapport; QSR, Quality of Social Response; SINT, Unusual Sensory Interest in PlayMaterial/Person; XINT, Excessive Interest in or References to Unusual or Highly Specific Topics or Objects or Repetitive Behaviors.

The factor structure of this model showed five factors that we named “Restricted, Repetitive Behaviors” (Stereotyped/Idiosyncratic use of words or phrases, Unusual Sensory Interest in PlayMaterial/Person, hand and finger and other complex mannerisms and Excessive Interest in or References to Unusual or Highly Specific Topics or Objects or Repetitive Behaviors), “Basic SOC_eye_” (Unusual Eye Contact [EYE] and Facial Expressions Directed to Examiner [EXPE]), “Basic SOC_ges_” (Descriptive, Conventional, Instrumental, orInformational Gestures [DGES] and Shared Enjoyment in Interaction [ENJ]), “Interaction Quality_reciprocity_” (Conversation [CONV], Amount of Reciprocal Social Communication [ARSC]), and Interaction Quality_appropriateness_” (QSR and OQR). “Basic SOC_eye_” correlated with “Basic SOC_ges_” (*r* = 0.94) and “Interaction Quality_conv_” correlated with “Interaction Quality_appropriateness_” (*r* = 0.92). All other factors were also intercorrelated as shown in a correlation matrix in Table 5.

**TABLE 5:** Factor correlation matrix for the final 5‐factor BEFA model (Bayesian exploratory factor analysis)

	5‐Factor model	
	Mean corr	sd
R:1:2	0.797	0.019
R:1:3	0.807	0.022
R:1:4	0.831	0.020
R:1:5	0.834	0.019
R:2:3	0.947	0.009
R:2:4	0.903	0.012
R:2:5	0.917	0.009
R:3:4	0.941	0.011
R:3:5	0.963	0.008
R:4:5	0.883	0.014

mean corr. = mean correlation of factors. sd = standard deviation.

### 4.3 Associations between ASD symptom dimensions and individual characteristics

In the **full sample** (ASD as well as non‐ASD), the RRB factor was negatively correlated with nonverbal IQ, meaning that individuals with higher IQ showed less repetitive behaviors. In the non‐ASD group this association was also found for verbal IQ. In the ASD group “RRB” was also negatively correlated to age – the older the child, the less repetitive behaviors were observed. Furthermore, in the ASD group but not in the non‐ASD group, “Interaction Quality” was negatively correlated with nonverbal and verbal IQ meaning that higher IQ was associated to less impaired interaction quality. The “Basic SOC” dimension showed only low and nonsignificant correlations with IQ. In the non‐ASD group higher age was associated with better “Basic SOC” skills. Sex was not correlated to any dimension in neither ASD nor non‐ASD individuals. Correlation coefficients are presented in Table 6. All coefficients represent small effects according to Cohen's interpretation of effect sizes. Correlations of the five BEFA dimensions fully resembled the pattern of correlation with the three symptom dimensions and are thus not further described.

**TABLE 6:** Associations between the ASD symptom dimensions and child characteristics for the three dimensions “Basic SOC”, “Interaction Quality” and “RRB”

	ASD		Non‐ASD	
	*r*	*d*	*r*	*d*
Basic SOC				
Age	−0.051	0.10	0.081^a^	0.16
Sex (male)	−0.047	0.09	−0.052	0.10
Verbal IQ	−0.025	0.05	0.000	0
Nonverbal IQ	−0.086	0.17	−0.120	**0.24**
Full scale IQ	−0.047	0.09	−0.012	0.02
Interaction quality				
Age	−0.042	0.08	−0.001	0.01
Sex (male)	−0.034	0.07	−0.036	0.07
Verbal IQ	−0.178^b^	**0.36**	−0.051	0.02
Nonverbal IQ	−0.185^b^	**0.38**	0.014	0.03
Full scale IQ	−0.184^b^	**0.38**	−0.017	0.03
RRB				
Age	−0.156^b^	**0.32**	−0.060	0.12
Sex (male)	−0.052	0.10	−0.008	0.02
Verbal IQ	−0.059	0.11	−0.088^a^	0.18
Nonverbal IQ	−0.160^b^	**0.33**	−0.139^b^	**0.28**
Full scale IQ	−0.123	**0.25**	−0.125^b^	**0.25**

^a^ significant on a 0.05 level.

^b^ significant on a 0.01 level.

*r* = Pearson correlation coefficient. *d* = Cohen's effect size (0.2–0.5 = small effect. 0.5–0.8 = medium effect. >0.8 = large effect). Bold numbers indicate effects exceeding the threshold of small effects.

Abbreviations: IQ, intelligence quotient; RRB, restricted, repetitive behaviors.

### 4.4 Associations with ASD diagnosis

The logistic regression model with the three dimensions (Basic SOC, interaction quality and RRBs) revealed an effect (χ^2^(3) = 767.8, *p* < 0.001) with satisfactory quality (Nagelkerkes *R* ^2^ = 0.59). All three dimensions contributed independently to the prediction of ASD versus non‐ASD diagnoses (RRBs: *B* = 0.87, OR = 2.39, *p* < 0.001, Basic SOC: *B* = 0.50, OR = 1.65, *p* < 0.001, interaction quality: *B* = 0.35 OR = 1.41, *p* < 0.001). All regression coefficients were positive, meaning that higher scores on all three dimensions were associated to an increased probability for an ASD diagnosis. Even after controlling for age and nonverbal IQ, the results are similar in that all three dimensions contribute independently to the prediction of an ASD or non‐ASD diagnosis.

A logistic regression with five factors (RRB, Basic SOC_eye_, Basic SOC_ges_, Interaction Quality_conv_ and Interaction Quality_observer_) was amended to investigate potential differential influences of the five BEFA factors. A significant effect (χ^2^(5) = 817.70, *p* < 0.001) with satisfactory quality (Nagelkerkes *R* ^2^ = 0.62) was observed. Four out of five factors contributed independently to the prediction of ASD versus non‐ASD diagnoses: RRBs: *B* = 0.95, OR = 2.59, *p* < 0.001, Basic SOC_eye_: *B* = 0.50, OR = 1.65, *p* < 0.001, Basic SOC_ges_: *B* = 0.53, OR = 1.69, *p* < 0.001, Interaction Quality_reciprocity_: *B* = 0.71, OR = 2.04, *p* < 0.001, and Interaction Quality_appropriateness_: *B* = 0.−0.11, OR = 0.89, *p* = 0.250.

Interaction Quality_appropriateness_ was the only factor that was not predictive of diagnosis.

## 5 DISCUSSION

The results of this replication study amended by a BEFA confirms that items from the ADOS‐2 diagnostic algorithm need to be parceled into at least two subdomains of social communication as suggested be Bishop et al. (2016). Our CFA replicated the previously reported observation of three latent dimensions in the ADOS algorithm reflecting “RRBs”, “Basic Social‐Communication” behaviors and “Interaction Quality” in fluently verbal individuals with ASD. However, a two‐dimensional model consisting of a single social‐communication factor and an RRB factor showed only marginally inferior fit indices. Furthermore, all three factors were highly interrelated and thus probably indicative of the same thing – that is, a diagnosis of ASD (or not). These large factor inter‐correlations likely signal unmodeled cross‐loadings (certain items are measuring more than one dimension) hinting at poor discriminant validity of the model. This suggests that the more parsimonious factor solution – with a single social‐communication factor should be preferred. To approach the problem of unmodeled cross‐loadings, a BEFA was conducted in the present study as it allows manifest variables to load only on a single factor but still permits extracted latent factors to be correlated. Using an uninformative prior and thus allowing the data to “paint the picture”, the BEFA model with the highest posterior probability comprised five factors. With regards to content, the five factor solution resembled in large the expected structure of the three‐dimensional model. The “Basic SOC” dimension was split into a factor comprising eye contact and facial expressions (EYE and EXPE) and another factor comprising gestures and shared enjoyment (DGES and ENJ). Both factors were highly correlated. The factor “Interaction Quality” was also divided in the current BEFA model with one pair of items measuring skills in conversation and the amount of reciprocal social communication (CONV and ARSC) representing more complex aspects of active social interaction relating to reciprocity. The other two items that belong to “Interaction Quality” are items that reflect complex aspects of social communication too but rather represent the observers evaluation of the appropriateness of the observed social behavior (OQR = Overall Quality of Rapport and QSR = Quality of Social Responses) and reflect the effort an examiner has to put into attempts to engage the child and into the social interaction overall (eliciting responses from the child).

As our findings show, the “Basic SOC” subdomain represents a replicable and stable factor. No associations of scores on basic social communication with child characteristics were found in the current sample which adds evidence to the argument of Bishop et al. that these basic impairments in nonverbal communication and shared affect seem quite specific for ASD and may be less affected by other phenotypical variables. Thus Basic SOC seem to be most appropriate as an index of ASD symptom severity. Both factors from the Basic SOC subdimension (Basic SOC_eye_, Basic SOC_ges_) contributed equally to the prediction of an ASD diagnosis.

Associations of “Interaction Quality” with child characteristics from the ASD group were restricted to IQ measures, no associations were found in the non‐ASD group. Unlike previous findings by Bishop et al. (2016) where scores on Interaction Quality were not predictive of an ASD diagnosis, all three dimensions of the ADOS algorithm (RRB, Basic SOC and Interaction Quality) contributed to the prediction of a diagnosis in the present sample. Although Interaction Quality made only a minor contribution it was still relevant. Following the split of the subdimension Interaction Quality into Interaction Quality_reciprocity_ and Interaction Quality_appropriateness_ from the BEFA, we found significant contributions to the ASD or non‐ASD diagnoses only for those complex aspects of interaction that relate to reciprocity (Conversation and Amount of Reciprocal Social Communication). Aspects of interaction quality that focus on the social appropriateness of communicative behavior (Overall Quality of Rapport and Quality of Social Responses) were not associated to child characteristics and did not contribute to the prediction of the diagnosis. This observation is tightly bound to the ADOS assessment however and may show a different pattern in different diagnostic instruments. One recent study identified latent factors from three different measures used to assess ASD symptoms and also found a two‐fold structure of an Interaction Quality factor relating to reciprocal interaction with adults on the one hand and peers on the other hand (Zheng et al., 2021). Together these findings suggest that the subdimension of “Interaction Quality” may be more complex and not yet sufficiently mapped by the investigated diagnostic instruments. All ADOS items related to the Interaction Quality dimension are summary items that are coded continuously throughout the ADOS‐2 evaluation. This leads to the high intercorrelation of all factors – in both CFA and BEFA analyses –indicating poor discriminative validity of the factors, as the items are per se not independent.

In order to separate the proposed subdimensions of social communication we need more objective (biological or behavioral) and independent markers. This may enable us to validate the specificity of impairments in basic nonverbal communication and shared affect for ASD and the value of information from measurements of interaction quality for differential diagnoses.

Most predictive for the diagnosis of ASD, was the presence of repetitive behaviors RRB in our sample. Our results indicate that restricted and repetitive behaviors are cardinal features of ASD in combination with impairments in Basic SOC and (less pronounced) Interaction Quality. This is in line with DSM‐5, where the presence of RRB is required for the diagnosis of ASD. Several studies demonstrated that the presence of RRBs increases the specificity of the diagnosis ASD (Jiujias, Kelley, & Hall, 2017; Kim & Lord, 2010). In sum, the current results add evidence to the notion that social communication impairments are multifaceted and that in order to characterize subtypes within the spectrum and in order to separate core and associated symptoms, a better understanding of different types of social communication impairment is needed.

### 5.1 Strengths and limitations

A key strength of this study is the considerable sample size and the inclusion of a clinical comparison group that comprises individuals with mental disorders that are relevant differential diagnoses to ASD. In consideration of the “replication crisis” (Lewandowsky & Oberauer, 2020) replicability is fundamental especially for research on the heterogeneous autism spectrum. Our presented results add substantial evidence to the proposed subdimensions of social‐communication impairment in ASD.

A limitation of the current study – as has been a limitation of the Bishop et al. study that we aimed to replicate – is its focus on verbally fluent individuals, excluding a large portion of individuals with ASD that are non‐verbal, younger and with intellectual disabilities. Whereas Basic Social Communication abilities were independent of age and IQ in our ASD sample, Interaction Quality and particularly RRBs were related to age and IQ. We thus need to expand future analyses to a broader sample, as studies of measurement invariance across developmental groups will improve our understanding of the latent structure of social communication deficits. Although we found no correlations of sex with any dimension in neither ASD nor non‐ASD individuals in the current sample, Bishop et al. did find associations of symptom dimensions with male sex. Particularly measures of interaction quality may vary with sex and further analyses are needed to investigate dimensions of social‐communication impairments in males and females separately.

## 6 CONCLUSION

Social communication is a multidimensional construct that can be influenced by individual, contextual, and other factors and thus requires a more precise approach (Bishop et al., 2016). By replicating previous findings in a large clinical sample our results support the efforts to further conceptualize the social‐communication impairments in ASD as two‐ or even multi‐dimensional. A better understanding of different types of social communication impairments will promote the identification of behaviorally relevant subgroups within ASD.

## CONFLICT OF INTEREST

The authors have declared that they have no competing or potential conflicts of interest.

## AUTHOR CONTRIBUTIONS

**Sanna Stroth:** Conceptualization; Data curation; Formal analysis; Investigation; Methodology; Project administration; Writing – original draft. **Hauke Niehaus:** Formal analysis; Methodology; Writing – review & editing. **Nicole Wolff:** Data curation; Investigation, Project administration. **Luise Poustka:** Funding acquisition; Resources; Writing – review & editing. **Veit Roessner:** Funding acquisition; Resources; Writing – review & editing. **Inge Kamp‐Becker:** Conceptualization; Funding acquisition; Project administration; Resources; Writing – original draft. **Dominik Endres:** methodology; writingReviewEditing.

## ETHICAL CONSIDERATIONS

Participants’ data were collected retrospectively from the respective medical record and analyzed anonymously. The procedure was approved by the local ethics committee (Az. 92/20) and due to the retrospective nature of data collection and analysis of anonymized data, the need for informed consent was waived by the ethics committee. All methods were performed in accordance with the relevant institutional and international research guidelines and regulations.

## Supporting information

Supporting Information S1

## Data Availability

The data that support the findings of this study are available on request from the corresponding author ‐ pending approval of the coauthors. The data are not publicly available due to privacy restrictions.
