# Supplementary material for: Subdimensions of social‐communication behavior in autism—A replication study
Source: JCPP Adv. 2022 May 4;2(2):e12077. doi: 10.1002/jcv2.12077 (PMC10242906; doi:10.1002/jcv2.12077)

**Table S1.** Model parameters and prior specification

| Parameter as used in “BayesFM” package | Value | Description |
| --- | --- | --- |
| Seed | 1500 | the random number generator in R |
| burnin | 10.000 | Burn-in period of the MCMC sampler. |
| iter | 50.000 | Number of MCMC iterations saved for posterior inference (after burn-in). |
| Nid | 2 | Minimum number of manifest variables dedicated to each latent factor for identification. |
| Kmax | 5 | Minimum and maximum number of latent factors. |
| HW-prior |  | Huand-Wand (2013) marginally noninformative prior on covariance matrix of the latent factors in the expanded model |
| kappa0,  xi0 | .5  .5 | First and second shape parameter of the Beta prior distribution on the probability $\tau_{0}$ that a manifest variable does not load on any factor |
| kappa | .1 | Concentration parameters of the Dirichlet prior distribution on the indicators. An uninformative Dirichlet prior was chosen, so each factor solution is a priori estimated as equally likey. |

**Table S2.** Abbreviations of the ADOS-2 algorithm items that entered the confirmatory factor analyses and Bayesian exploratory factor analysis (CFA and BEFA)

| STER | Stereotyped/Idiosyncratic Use Of Words or Phrases |
| --- | --- |
| CONV | Conversation |
| DGES | Descriptive, Conventional, Instrumental, or Informational Gestures |
| EYE | Unusual Eye Contact |
| QSOV | Quality of Social Overtures |
| EXPE | Facial Expressions Directed to Examiner |
| ENJ | Shared Enjoyment in Interaction |
| QSR | Quality of Social Response |
| ARSC | Amount of Reciprocal Social Communication |
| OQR | Overall Quality of Rapport |
| SINT | Unusual Sensory Interest in Play Material/Person |
| MAN | Hand and Finger and Other Complex Mannerisms |
| XINT | Excessive Interest in or References to Unusual or Highly Specific Topics or Objects or Repetitive Behaviors |

**Figure S1**. CFA models with two (left) and three (right) latent dimensions. basic = Basic Social Communication; interaction = Interaction Quality and rrb = Restricted and Repetitive Behaviors.


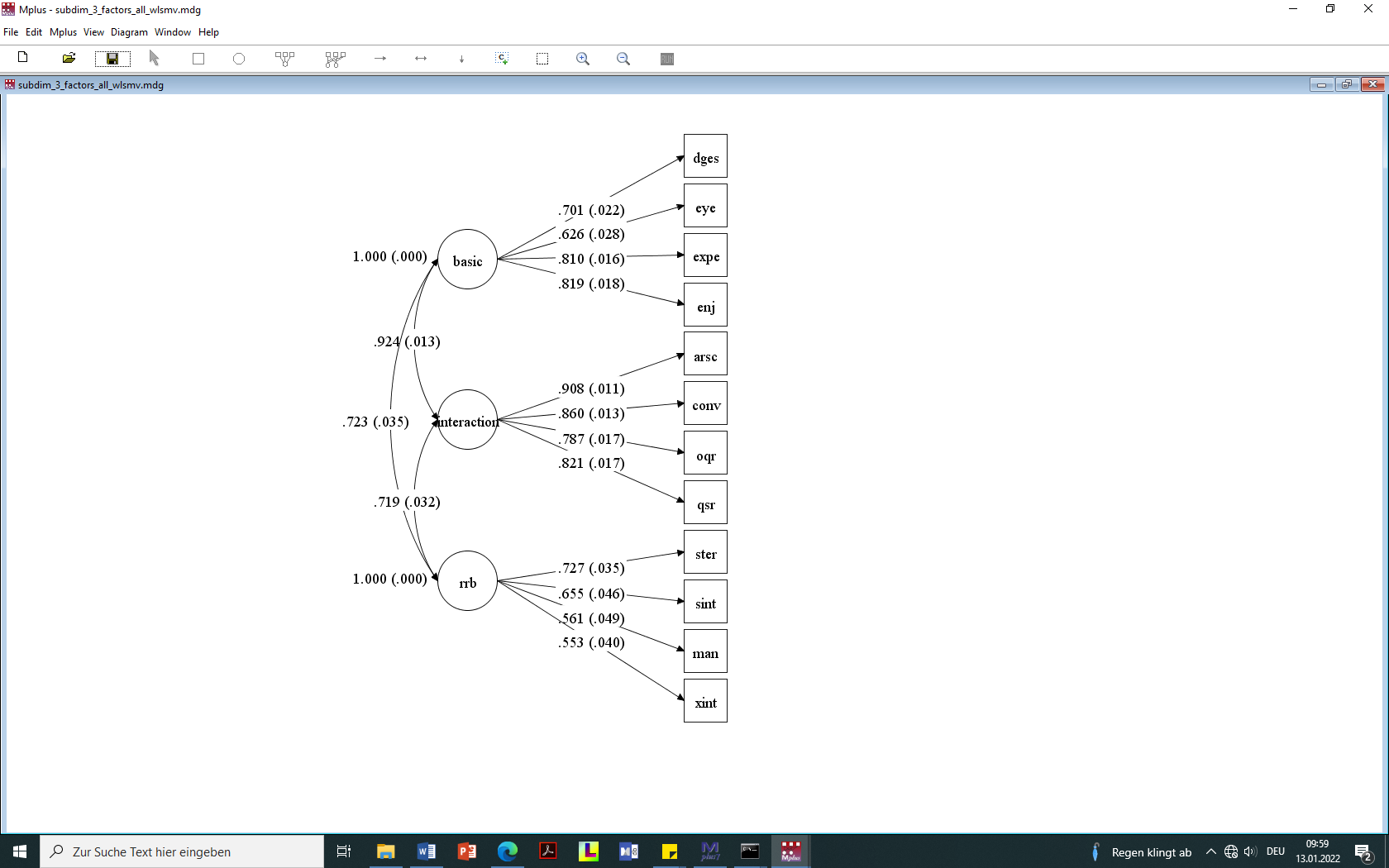

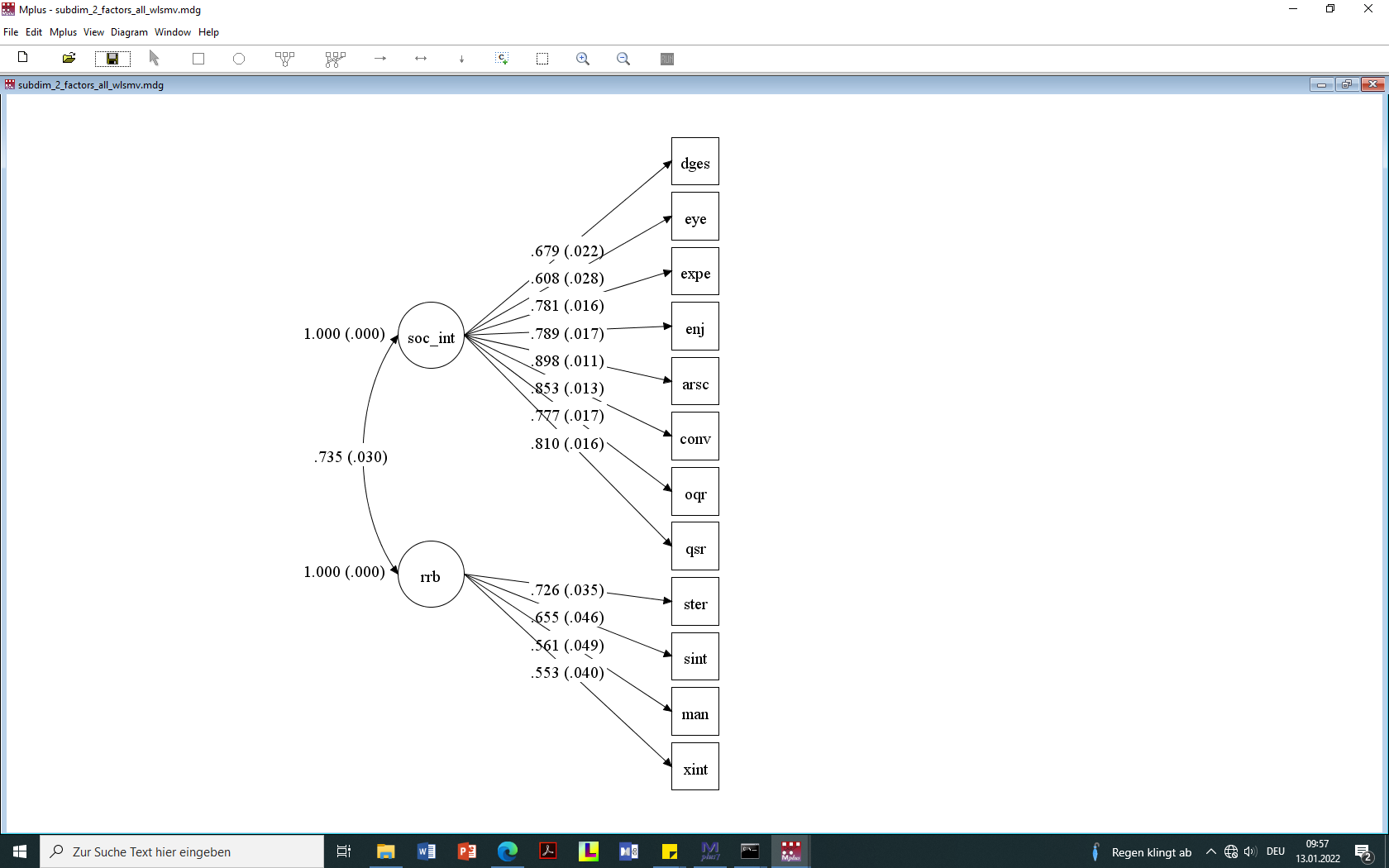

Supplement: Supplementary file 1 — Supporting Information S1 [file JCV2-2-e12077-s001.docx]
